# Feasibility of a novel atrioventricular delay optimization method using transmitral and pulmonary venous flow in patients with sequential ventricular pacing or cardiac resynchronization therapy

**DOI:** 10.1007/s12574-014-0237-x

**Published:** 2014-12-19

**Authors:** Kenzo Fukuhara, Hiroyuki Okura, Terumasa Koyama, Teruyoshi Kume, Yoji Neishi, Akihiro Hayashida, Kiyoshi Yoshida

**Affiliations:** Division of Cardiology, Kawasaki Medical School, Kurashiki, Japan

**Keywords:** Doppler echocardiography, Left ventricular function, Pacemaker, Cardiac resynchronization therapy, AV delay

## Abstract

**Background:**

Although several echo-Doppler methods were proposed to optimize atrioventricular (AV) delay in patients with sequential ventricular pacing, “echo-guided” AV optimization has not been widely adopted clinically. A combination of trasmitral flow (TMF) and pulmonary venous flow (PVF) measurements may be beneficial to further optimize AV delay to achieve better cardiac function. The aim of this study was to assess the feasibility and usefulness of AV delay optimization by combined use of TMF and PVF.

**Methods:**

A total of 32 patients after sequential ventricular pacemaker implantation were enrolled and studied. The optimal AV delay was defined as the timing to minimize the duration between PVF reversal (a) wave and the duration of the “A” wave of TMF. Stroke volume was measured at the “optimized” AV delay (AVD_OPT_) and was compared with that obtained at shorter (AVD_OPT_ − 50 ms) and longer (AVD_OPT_ + 50 ms) AV delays.

**Results:**

AV optimization was feasible in 27 of 32 patients (87 %). Stroke volume at AVD_OPT_ was significantly higher than that at shorter or longer AV delay (63 ± 18 ml vs. 57 ± 15 ml vs. 56 ± 16 ml, *P* = 0.001).

**Conclusions:**

AV delay optimization using TMF and PV flow was feasible. Usefulness of this method requires further investigation with a larger study population.

## Introduction

Left ventricular (LV) dysfunction may develop as a result of LV dyssynchrony and/or inappropriate atrioventricular (AV) delay in some patients after single chamber, ventricular pacing. Even after dual-chamber sequential pacing, maintenance of AV synchrony is necessary to preserve cardiac function and to achieve a better prognosis [1, 2]. AV delay optimization is, therefore, important to maintain better cardiac function and a favorable long-term outcome after sequential pacing [3, 4] or cardiac resynchronization therapy [5, 6]. Although several echo-Doppler- [7–13] as well as electrocardiogram- [14–17] based methods to optimize AV interval have been proposed, routine or systematic use of AV optimization remains controversial [5, 6, 18–20]. Transmitral flow (TMF) by transthoracic Doppler echocardiography is commonly used to optimize AV delay. However, the advantage of echo-Doppler-based AV optimization over fixed AV delay or a commercially available AV optimization algorithm based on electrocardiogram has not been proven yet.

Theoretically (based on the Frank–Starling law), AV delay should be optimized to achieve maximal LV filling without deterioration of LV function [2]. Because TMF alone does not reflect both systolic function and LV filling pressure, TMF-based AV optimization may not provide enough advantage over the other methods. A previous echo-Doppler study demonstrated that the difference between the duration of pulmonary venous flow reversal (PVa) and mitral forward flow during atrial systole (A) reliably estimates LV filling pressure [21]. We hypothesized that a combination of TMF and PV flow measurements may be beneficial to further optimize AV delay to achieve better cardiac function with adequate LV filling pressure. Therefore, the aim of this study was to assess feasibility of the AV delay optimization by combined use of TMF and PV flow.

## Materials and methods

### Study population

This study included 32 patients after dual-chamber pacing for complete AV block (*n* = 26, mean age = 79 ± 8 years; 12 males) or cardiac resynchronization therapy (*n* = 6, mean age = 65 ± 16 years; 4 males). The exclusion criteria were current atrial arrhythmia and frequent premature ventricular beats. Informed consent was provided by each participant before enrollment in this study.

### Study protocol

Echocardiography was performed with a Sonos 5500 and S3 transducer (Philips Medical Systems, Andover, MA, USA). TMF was obtained from apical 3-chamber or 4-chamber views with the sample volume positioned at the tip of the mitral leaflets. TMF consists of 2 distinct flow signals, early or E wave and late or A wave during atrial contraction. PV flow was obtained from an apical 4-chamber view with the sample volume placed in the left superior pulmonary vein. An effort was made to maintain the same position of the pulsed Doppler sample throughout the echo-Doppler examination.

**Fig. 1:**
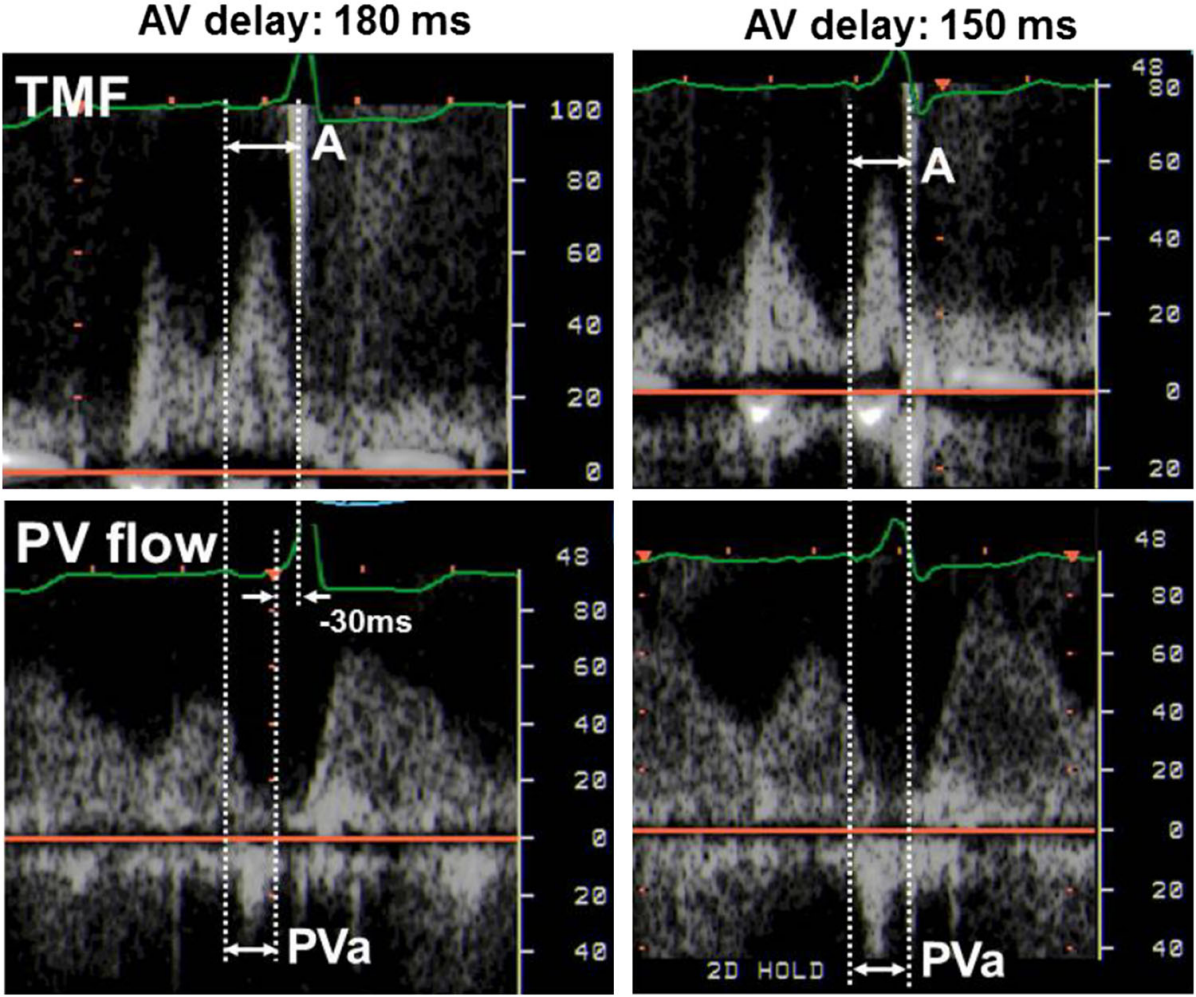
AV delay optimization using TMF and PV flow. **a** (Step 1) At a pre-set AV delay (=180 ms in this case), both TMF and PV flow signal were recorded. (Step 2) The difference in duration between PVa and A wave was measured (=−30 ms). **b** (Step 3) Optimal AV delay was calculated as (pre-set AV delay) + (duration of PVa − duration of A). In this case, the optimal AV delay was calculated as 180 ms + (−30 ms) = 150 ms

AV delay optimization was performed using TMF and PV flow at rest. Optimal AV delay (AVD_OPT_) was defined as the AV delay where the duration of PVa minus A was the minimum (=0). Because the onset of the A wave cannot be always detected, the difference between the duration of PVa and A was alternatively measured as (time interval between the onset of the Q wave and the end of the A wave) − (time interval between the onset of the Q wave and the end of the PVa wave). To simplify this method, TMF and PV flow were recorded at the pre-set AV delay. Then, AVD_OPT_ was determined as (pre-set AV delay) + (duration of PVa − duration of A) (Fig. 1). Stroke volume (SV) was measured by a pulsed Doppler method obtained at the LV outflow tract and was used as an index to assess cardiac function during AV optimization. SV obtained at the AVD_OPT_ was compared with SV obtained at shorter (AVD_OPT_ − 50 ms) or longer (AVD_OPT_ + 50 ms) AV delays.

### Statistical analysis

The measurements are expressed as mean ± standard deviation. Statistical analyses were performed with one-way analysis of variance (ANOVA) using the Bonferroni post hoc test. Values of *P* < 0.05 were considered statistically significant.

## Results

Clinical characteristics of the 32 patients are shown in Table 1. Dual chamber (DDD) pacing was used in 12 patients and ventricular (VDD) pacing in 20 patients. All patients after dual-chamber sequential pacing were in New York Heart Association (NYHA) class I or П. On the other hand, all patients after cardiac resynchronization therapy (CRT) were in NYHA class III. Left ventricular ejection fraction (LVEF) was 55 ± 15 %.

**Table 1:** Clinical characteristics

	(*n* = 32)
Age (years)	76 ± 11
Male gender, *n* (%)	16 (50)
DDD/VDD	12/20
Pacemaker/CRT	6/26
Ischemic heart disease, *n* (%)	9 (28)
Diabetes, *n* (%)	12 (38)
Hypertension, *n* (%)	20 (62)
Dyslipidemia, *n* (%)	14 (44)
NYHA class (I/II/III/IV)	24/2/6/0
Medication, *n* (%)	
β-blockers	10 (31)
ACE-I/ARB	18 (56)
Loop diuretics	11 (34)
Spironolactone	6 (19)
Digitalis	1 (3)
Statins	12 (38)

*ACE-I* angiotensin-converting enzyme inhibitors, *ARB* angiotensin receptor blockers, *CRT* cardiac resynchronization therapy

**Fig. 2:**
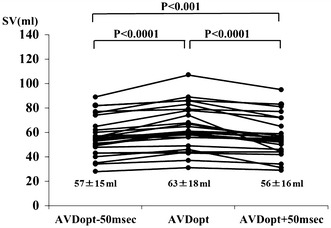
Comparison of SV in all patients. SV obtained at the AVD_OPT_ was compared with SV obtained at shorter (AVD_OPT_ − 50 ms) or longer (AVD_OPT_ + 50 ms) AV delays

AV optimization using our current method could be performed in 27 of 32 patients (84 %). In the remaining 5 patients, adequate PV flow signal could not be recorded. The measurements made in all patients are summarized in Tables 2 and 3. Mean AVD_OPT_ was 143 ± 35 ms. As expected, the mean AVD_OPT_ was significantly lower in the VDD than in the DDD mode (133 ± 32 ms vs. 170 ± 37 ms, *P* = 0.014). SV at AVD_OPT_ was significantly higher than shorter or longer AV delay (63 ± 18 ml vs. 57 ± 15 ml vs. 56 ± 16 ml, *P* = 0.001) (Fig. 2).

**Table 2:** Hemodynamic and Doppler echocardiography parameters

Patient no.	Gender	Age	Disease	LVEF (%)	LVDd (mm)	LVDs (mm)	Ao TVI (cm)	SV (ml)
1	M	75	Complete AV block	60	48	30	22	53
2	F	46	Complete AV block	60	48	30	22	61
3	F	81	Complete AV block	48	46	37	12	43
4	M	73	Complete AV block	58	42	26	23	86
5	F	84	Complete AV block	54	41	32	21	49
6	M	75	Complete AV block	59	51	31	26	79
7	F	88	Complete AV block	62	43	23	30	61
8	F	77	Complete AV block	58	43	29	20	44
9	M	73	Complete AV block	54	44	32	34	108
10	M	78	Complete AV block	58	42	28	17	54
11	M	80	Complete AV block	65	42	24	21	83
12	M	81	Complete AV block	56	36	21	15	31
13	F	82	Complete AV block	63	42	26	26	55
14	M	78	Complete AV block	55	54	36	19	52
15	M	83	Complete AV block	71	45	20	55	74
16	M	81	Complete AV block	71	43	30	26	81
17	F	82	Complete AV block	67	50	33	20	56
18	F	90	Complete AV block	75	32	17	18	35
19	F	79	Complete AV block	67	37	19	12	26
20	F	72	Complete AV block	67	47	29	29	89
21	F	82	Complete AV block	61	48	28	26	68
22	M	74	Complete AV block	54	54	33	19	65
23	M	38	DCM	36	70	58	15	61
24	M	75	Ischemic heart disease	32	61	50	21	78
25	F	84	Ischemic heart disease	76	34	16	33	44
26	M	64	DCM	45	57	39	20	60
27	M	71	DCM	31	63	60	20	56

*LVEF* left ventricular ejection fraction; *LVDd* left ventricular end-diastolic diameter; *LVDs* left ventricular end-systolic diameter; *Ao TVI* aorta time velocity integral; *SV* stroke volume; *DCM* dilated cardiomyopathy

**Table 3:** Pacing mode, pacing rate at initial enrollment and TMF A, PVa duration pre and post AV delay optimization

Patient no.	Pacing mode	HR	Pre AV delay optimization		Post AV delay optimization	
			TMF A duration	PVa duration	TMF A duration	PVa duration
1	DDD (A sense V pace)	70	115	120	130	130
2	VDD (A sense V pace)	72	145	140	145	140
3	DDD (A sense V pace)	60	165	155	165	155
4	DDD (A sense V pace)	60	160	160	160	160
5	VDD (A sense V pace)	70	150	125	150	125
6	VDD (A sense V pace)	60	120	130	120	130
7	VDD (A sense V pace)	75	170	100	122	122
8	VDD (A sense V pace)	70	115	130	126	122
9	VDD (A sense V pace)	55	145	145	145	145
10	VDD (A sense V pace)	60	140	150	155	145
11	DDD (A sense V pace)	60	165	165	165	165
12	VDD (A sense V pace)	62	135	160	135	160
13	VDD (A sense V pace)	70	135	110	150	135
14	DDD (A sense V pace)	69	150	145	115	125
15	VDD (A sense V pace)	60	135	115	135	115
16	VDD (A sense V pace)	60	175	180	185	190
17	VDD (A sense V pace)	60	145	105	135	130
18	VDD (A sense V pace)	75	180	140	130	130
19	VDD (A sense V pace)	70	130	125	115	115
20	VDD (A sense V pace)	60	140	130	140	130
21	VDD (A sense V pace)	76	110	125	110	125
22	DDD (A pace V pace)	80	150	115	150	115
23	DDD (A sense V pace)	75	87	75	–	–
24	VDD (A sense V pace)	65	–	–	–	–
25	DDD (A pace V pace)	60	127	–	–	–
26	DDD (A pace V pace)	60	–	–	–	–
27	VDD (A sense V pace)	65	150	145	150	145
28	DDD (A sense V pace)	70	140	170	140	170
29	VDD (A sense V pace)	96	130	125	130	125
30	VDD (A sense V pace)	80	150	170	130	145
31	DDD (A pace V pace)	60	115	155	115	155
32	VDD (A sense V pace)	60	–	–	–	–

*TMF* transmitral flow

**Fig. 3:**
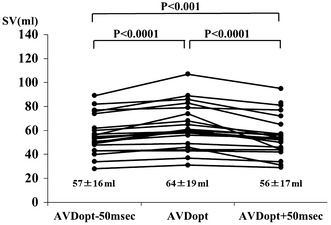
Comparison of SV in patients after sequential, dual chamber pacing for complete AV block. SV obtained at the AVD_OPT_ was compared with SV obtained at shorter (AVD_OPT_ − 50 ms) or longer (AVD_OPT_ + 50 ms) AV delays

In a subset of patients after sequential dual-chamber pacing for complete AV block, AV optimization could be performed in 22 of 26 patients (85 %). The AVD_OPT_ in VDD mode was 128 ± 38 ms and the AVD_OPT_ in the DDD pacing mode was 177 ± 39 ms. SV with AVD_OPT_ was significantly higher than shorter or longer AV delay (64 ± 19 ml vs. 57 ± 16 ml vs. 56 ± 17 ml, *P* = 0.0001) (Fig. 3).

**Fig. 4:**
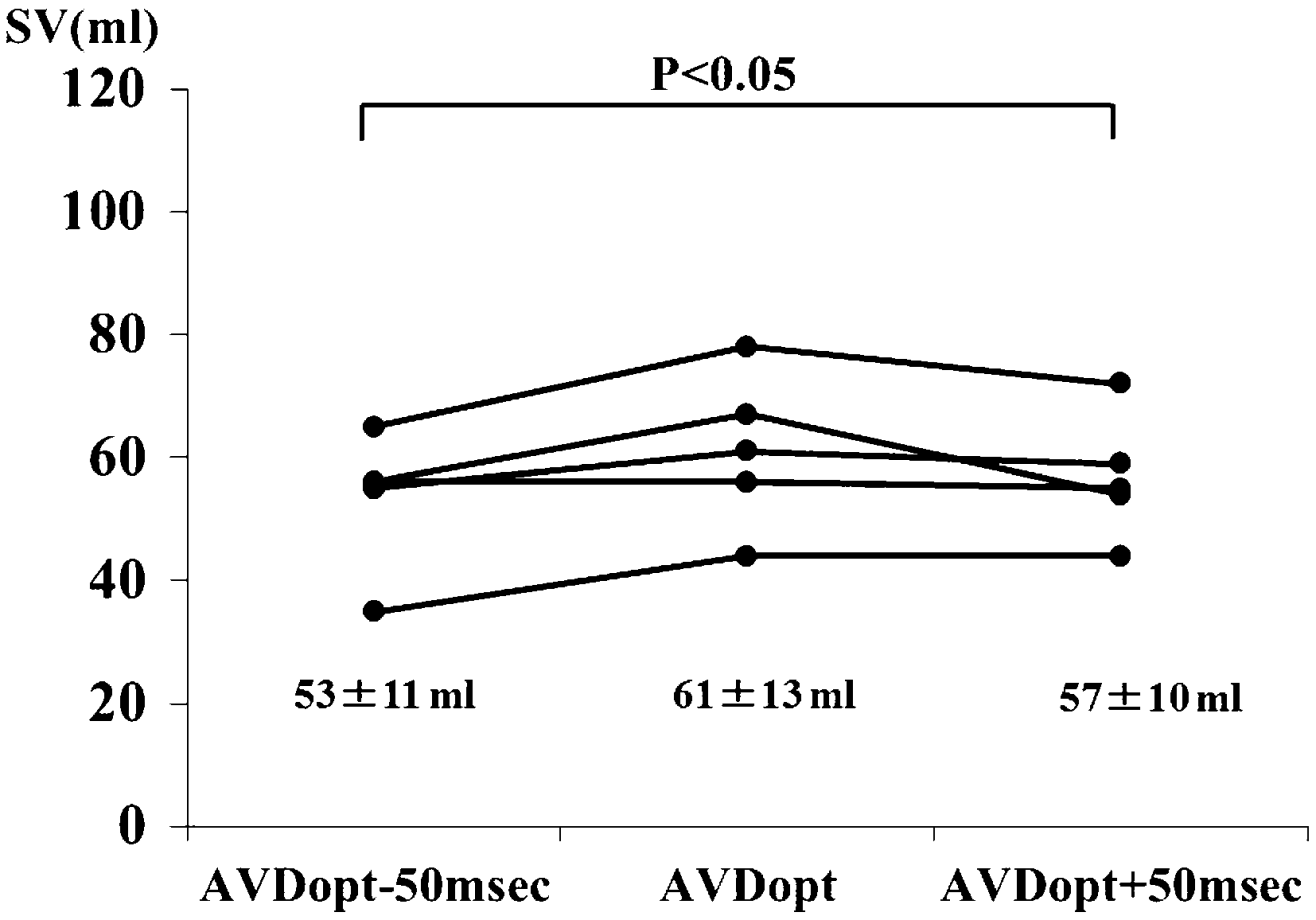
Comparison of SV in CRT recipients. SV obtained at the AVD_OPT_ was compared with SV obtained at shorter (AVD_OPT_ − 50 ms) or longer (AVD_OPT_ + 50 ms) AV delays

Similarly, in a subset of patients after CRT, AV optimization could be performed in 5 of 6 patients (83 %). The AVD_OPT_ in VDD mode was 128 ± 38 ms and the AVD_OPT_ in the DDD pacing mode was 177 ± 39 ms. SV with AVD_OPT_ was significantly higher than shorter or longer AV delay (61 ± 13 ml vs. 53 ± 11 ml vs. 57 ± 10 ml, *P* = 0.026) (Fig. 4).

Reproducibility of PV flow measurements was analyzed. Correlation coefficients were high for repeated measurements by the same observer (*r* = 0.98 for duration of PVa minus A) and measurements by 2 different observers (*r* = 0.88 for duration of PVa minus A).

## Discussion

This study shows that AV delay optimization based on a new echo-Doppler method using TMF and PV flow is feasible. In addition, increased SV during AV delay optimized by this method may suggest a potential favorable impact on cardiac function and possibly prognosis.

A previous randomized, prospective study comparing echo-guided AV delay optimization and an empiric, fixed AV delay of 120 ms demonstrated improved clinical outcome at 3 months in patients with echo-guided AV optimization [19]. In their study, optimal AV delay was defined as the largest aortic velocity–time integral at one of eight tested AV intervals (between 60 and 200 ms). On the other hand, a more recent large-scale randomized prospective multicenter trial (SMART-AV trial) to compare between a fixed empirical AV delay (120 ms), echocardiographically optimized AV delay, and AV delay optimized with SmartDelay electrocardiogram-based algorithm did not show superiority of echocardiography or SmartDelay over a fixed AV delay of 120 ms [18]. In their study, Ritter’s method [10, 22] and/or an iterative method [23] using TMF were used to optimize AV delay as endorsed by the American Society of Echocardiography [23, 24]. Based on their negative results, the authors stated that routine echocardiographic AV optimization using the American Society of Echocardiography recommended method for patients with CRT should be abandoned [18]. However, it is not certain whether all echo-Doppler methods should be abolished.

Ritter et al. [22] first reported an echo-Doppler method to optimize AV delay in patients with complete AV block and a normal LV systolic function. Ritter et al. defined the AV delay with the echo method that provided the longest diastolic filling time without interruption of the A wave. Ritter’s formula, which can be regarded as the current “gold standard” in AV delay optimization [24] requires 2 measurements: (1) QA short = the time interval between the onset of the Q wave and the end of the truncated “A” wave of the TMF at a short (30–60 ms) AV delay; and (2) QA long = the time interval between the onset of the Q wave and the end of the “A” wave of the TMF at a long (200 ms) AV interval. According to the formula, optimal AV delay was calculated as AV long − (QA short − QA long). This method has been used in several clinical trials because it is a simple, non-invasive and reproducible method [20]. On the other hand, Ishikawa et al. used diastolic mitral regurgitation to optimize AV delay. As compared with Ritter’s method in which AV delay was optimized to achieve the highest cardiac output, Ishikawa’s method is to achieve the lowest possible left atrial or LV filling pressure [9, 25]. In our present study, we used both TMF and PV flow to achieve the lowest LV filling pressure and the highest SV.

The concept of Doppler assessment of LV filling pressure using both TMF and PV flow was first reported in 1993 by Rossvoll and Hatle [21]. The difference in duration between PVa of the PV flow and antegrade A wave by the TMF was positively and strongly correlated with LV end-diastolic pressure (*r* = 0.68, *P* < 0.001). A longer duration of PVa versus A wave predicted increased (>15 mmHg) LV end-diastolic pressure [21]. The mechanisms for a longer duration of PVa than the A wave was explained by the increased LV end-diastolic pressure as a result of reduced LV compliance. Therefore, an AV delay that does not prolong PVa more than the A wave could be considered as a hemodynamically optimal AV delay. Although our preliminary data suggest that AV optimization based on the TMF and PV flow is feasible, it was not possible for AV optimization to be performed in some patients in whom PV flow could not be detected. This is a possible limitation of this study. Detection of the PV flow signal using the transthoracic approach depends upon the image quality of the echo-Doppler machine. Although the sensitivity of the Doppler measurements for some specific conditions was not sufficient when using old echo-Doppler machines and therefore required contrast enhancement [26, 27], modern echo-Doppler machines have sufficiently sensitive Doppler equipment [28, 29]. Another apparent limitation is that 2 different Doppler measurements are required for our method which appears to be time consuming. However, as compared with Ritter’s method, which requires 2 TMF recordings at 2 different AV delay settings, our method is less time consuming.

Because this is a small pilot study, further investigations will be necessary. First, the hemodynamically favorable acute results should be confirmed by invasive hemodynamic monitoring. Second, the long-term clinical impact of the acute results should be investigated by a serial observation of the study population. Finally, the advantages of the current method should be investigated by comparing it with other echo-Doppler methods or empirical fixed AV delay prospectively.

## Conclusions

A novel AV delay optimization method using TMF and PV flow has been shown to be feasible. The usefulness of this method requires further investigation with a larger study population.
